# Metabolite profiling of somatic embryos of *Cyclamen persicum* in comparison to zygotic embryos, endosperm, and testa

**DOI:** 10.3389/fpls.2015.00597

**Published:** 2015-08-04

**Authors:** Traud Winkelmann, Svenja Ratjens, Melanie Bartsch, Christina Rode, Karsten Niehaus, Hanna Bednarz

**Affiliations:** ^1^Institute of Horticultural Production Systems, Leibniz Universität HannoverHannover, Germany; ^2^Faculty of Biology, Bio 27, Proteome and Metabolome Research, Bielefeld UniversityBielefeld, Germany

**Keywords:** *in vitro* propagation, proline, ornamental plant, seeds, somatic embryogenesis

## Abstract

Somatic embryogenesis has been shown to be an efficient *in vitro* plant regeneration system for many crops such as the important ornamental plant *Cyclamen persicum*, for which this regeneration pathway of somatic embryogenesis is of interest for the vegetative propagation of parental lines as well as elite plants. However, somatic embryogenesis is not commercially used in many crops due to several unsolved problems, such as malformations, asynchronous development, deficiencies in maturation and germination of somatic embryos. In contrast, zygotic embryos in seeds develop and germinate without abnormalities in most cases. Instead of time-consuming and labor-intensive experiments involving tests of different *in vitro* culture conditions and plant growth regulator supplements, we follow a more directed approach. Zygotic embryos served as a reference and were compared to somatic embryos in metabolomic analyses allowing the future optimization of the *in vitro* system. The aims of this study were to detect differences in the metabolite profiles of torpedo stage somatic and zygotic embryos of *C. persicum*. Moreover, major metabolites in endosperm and testa were identified and quantified. Two sets of extracts of two to four biological replicates each were analyzed. In total 52 metabolites were identified and quantified in the different tissues. One of the most significant differences between somatic and zygotic embryos was that the proline concentration in the zygotic embryos was about 40 times higher than that found in somatic embryos. Epicatechin, a scavenger for reactive oxygen species, was found in highest abundance in the testa. Sucrose, the most abundant metabolite was detected in significantly higher concentrations in zygotic embryos. Also, a yet unknown trisaccharide, was significantly enriched in zygotic embryos.

## Introduction

Since its first description in carrot in Reinert (1958), and Steward et al. (1958), somatic embryogenesis, i.e., the regeneration of bipolar structures from somatic cells undergoing a development similar to zygotic embryos, has been described for a high number of plant species. This *in vitro* regeneration system offers several advantages such as high efficiency, origin in a single or only few cells, up-scaling by usage of liquid culture systems and the possibility to store the propagules after desiccation and encapsulation as artificial seeds. Thus, somatic embryogenesis is not only of interest for plant propagation, but also for genetic transformation. However, the potential of somatic embryogenesis has not been tapped in most plant species, except for *Pinus* species which are commercially propagated via this regeneration pathway (Cyr and Klimaszewska, 2002). For the majority of species, severe limitations have been reported like asynchronous development of somatic embryos, malformations such as fused cotyledons or developmental abnormalities, loss of embryogenic potential after a short time of subculturing, pronounced genotypic differences in regeneration efficiency, low germination rates due to the absence of a maturation phase, and somaclonal variation.

Cyclamen (*Cyclamen persicum* MILL.) is an important horticultural crop which is sold as an ornamental pot plant in high numbers, with an annual production estimated at about 150–200 million plants worldwide (Schwenkel, 2001). The current way of propagation by seeds involves high costs for seed production due to manual emasculation and pollination, and heterogeneity in many cultivars. Therefore, there is a high level of interest in vegetative propagation for clonal multiplication of parental genotypes of F_1_ hybrid cultivars, but also for mass propagation of elite plants. Whereas propagation by cuttings is not applicable in this tuber forming plant, somatic embryogenesis has been described for cyclamen by several groups (e.g., Wicart et al., 1984; Otani and Shimada, 1991, Kiviharju et al., 1992; Kreuger et al., 1995; Takamura et al., 1995; Schwenkel and Winkelmann, 1998, reviewed by Jalali et al., 2012). In our system embryogenic callus is induced from somatic cells in ovules using 2,4-D (2,4-dichlorophenoxyacetic acid) containing medium (Winkelmann, 2010). This embryogenic callus can be propagated on solid medium as well as in liquid culture (Winkelmann et al., 1998), while the transfer to plant growth regulator-free medium initiates differentiation. Within 4 weeks, torpedo-shaped somatic embryos develop which can be singularized for germination (Winkelmann, 2010). Several problems were observed during somatic embryogenesis in cyclamen, like asynchronous development (Rode et al., 2011), precocious germination, lack of desiccation tolerance, or the absence of a growth arrest connected to maturation (Schmidt et al., 2006).

In order to overcome these limitations, a better understanding of zygotic embryogenesis with special emphasis on the surrounding tissue shaping the zygotic embryo, the endosperm, is pursued. Knowledge about the physical and biochemical conditions in seeds during their development shall be used to mimic these conditions in the tissue culture system. By comparing *C. persicum* somatic and zygotic embryos on the transcriptomic level, Hoenemann et al. (2010) found higher levels of expression of genes involved in oxidative stress response in somatic embryos. On the proteomic level (Winkelmann et al., 2006; Bian et al., 2010; Rode et al., 2011) enzymes of the carbohydrate metabolism, heat shock proteins and a glutathione-*S*-transferase were observed to be more abundant in somatic embryos, again pointing to differences in stress response of both types of embryos. The proteomic analysis of the endosperm of *C. persicum* (Mwangi et al., 2013) revealed the accumulation of storage proteins of the 7S globulin type and proteins involved in synthesis of other storage compounds, i.e., lipids and xyloglucans. Metabolomic comparisons of somatic and zygotic embryos or primary metabolite profiles of the endosperm have not been studied so far for cyclamen.

The aims of this study were to compare somatic embryos of *C. persicum* in the torpedo stage to zygotic embryos in a comparable morphological stage regarding their metabolite profiles. Moreover, testa and endosperm were also included in these metabolite analyses, since the endosperm not only provides nutrition but also important signaling stimuli that regulate the development of the embryo (Linkies et al., 2010). In future studies similar analyses need to be performed throughout the development of zygotic embryos to gain insights into the embryogenesis process. From the results, we expect to enable the optimization of somatic embryogenesis by adjusting the culture media and the physical growth conditions.

## Materials and Methods

### Plant Material

The commercial F_1_ hybrid cultivar ‘Maxora Light Purple’ (breeder: Varinova BV, Berkel en Rodenrijs, The Netherlands) was used in this study. A single genotype of this cultivar had been vegetatively propagated by somatic embryogenesis, resulting in embryogenic cultures for the generation of somatic embryos and in clonally multiplied greenhouse grown plants serving as donor plants for the seed tissues.

#### Seed Tissues

**FIGURE 1:**
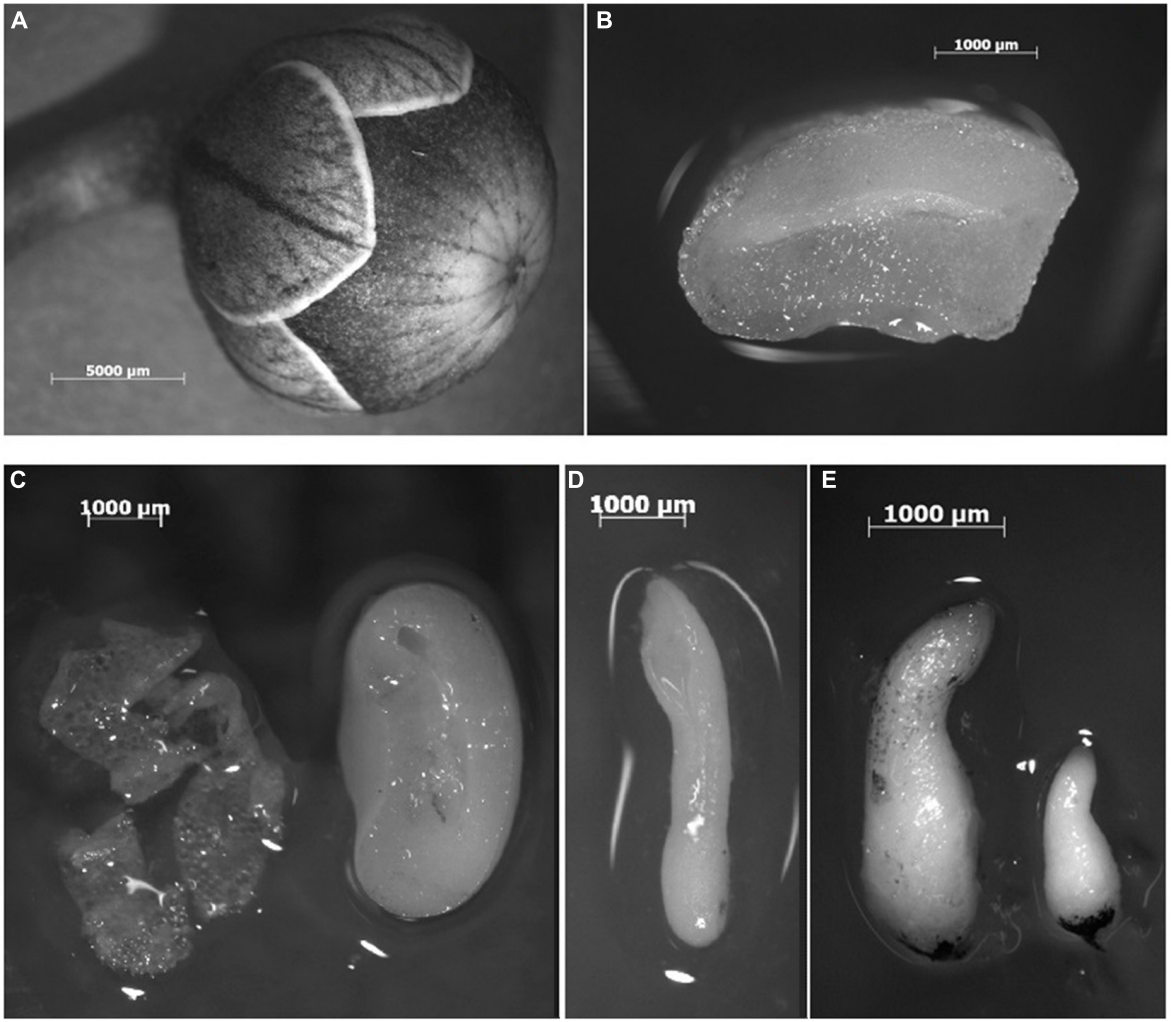
**Plant materials submitted to the metabolite analyses of *Cyclamen persicum* ‘Maxora Light Purple.’ (A)** Seed capsule 11 WAP, **(B)** cross section of a seed 11 WAP, **(C)** testa (left) and endosperm (right) prepared from a seed 11 WAP, **(D)** zygotic embryo dissected from a seed 11 WAP, **(E)** torpedo stage somatic embryos after 4 weeks differentiation.

Flowering plants were cultivated in a climatized greenhouse at 18°C and with supplemental light (16 h, 12 klx supplied by Philips SON T lamps) in the winter months. On four days per week plants were fertilized with 0.07% Universol Blau (18-11-18+2.5 MgO+trace elements; Everris, Nordhorn, Germany), while on one day they received 0.2% Hakaphos Rot (18-12-24+4 MgO+ trace elements; COMPA expert GmbH, Münster, Germany). Plant protection was applied according to horticultural practice. Closed flower buds were emasculated, the anthers were dried overnight for pollen release and after 24 h pollination was carried out. Eleven weeks after pollination (11 WAP), seed capsules (**Figure 1A**) were harvested and seeds (**Figure 1B**) were dissected under the microscope into the three tissues to be analyzed: testa, endosperm, and zygotic embryos in the torpedo stage (**Figures 1C,D**). Samples were mixtures of material of several seed capsules from different plants each and separation of the tissue types was possible without cross-contaminating the samples. All plant materials were immediately frozen in liquid nitrogen and freeze-dried after short storage at -80°C.

In 2012, three biological replicates of all tissues were analyzed, whereas in 2014 three biological replicates were used for endosperm and zygotic embryos and four for testa and somatic embryos. However, the chromatogram of one replicate from zygotic embryos in 2014 could not be evaluated.

#### Somatic Embryos

Somatic embryos were harvested following the protocol described in detail in Schwenkel and Winkelmann (1998), Winkelmann et al. (1998), and Winkelmann (2010). Briefly, embryogenic callus was induced from ovules on modified half-strength MS (Murashige and Skoog, 1962) medium containing 9.05 μM 2,4-D and 3.94 μM 2iP (6-(γ,γ-dimethylallylamino)purine) and propagated in suspension cultures. For differentiation of somatic embryos, the cell fraction 500–1000 μm was adjusted to a density of 10% packed cell volume, and 1 ml was plated onto 9 cm Petri dishes each containing 20 ml of plant growth regulator-free half-strength MS medium solidified with 4 g l^-1^ gelrite (Duchefa, Haarlem, Netherlands). After 4 weeks of culture at 24°C in the dark, about 80–100 torpedo-shaped embryos (**Figure 1E**) were selected per biological replicate (three in 2012 and four in 2014) and immediately transferred to liquid nitrogen.

### Metabolite Extraction

About 5 mg of lyophilized samples were homogenized using a Ribolyzer (3 × 45 s, 6.5 m/s) with 0.5 g of silica beads (0.5 mm) and 1 ml 80% methanol containing 10 μM ribitol as internal standard. The dry weight of all samples was determined and documented for later normalization. The extract was centrifuged at 18 400 g for 30 min. 750 μl of supernatant was dried in a stream of nitrogen gas and derivatized by 100 μl methoxylamin-hydrochloride (20 mg ml^-1^ in pyridine, Sigma-Aldrich) for 90 min and 100 μl *N*-methyl-*N*-trimethylsilyl-trifluoroacetamide (MSTFA, Macherey-Nagel) for 30 min with alkane retention time indices [C12, C15, C18, C19, C22, C28, C32 (each 0.55 mg ml^-1^ final concentration) and C36 (1.1 mg ml^-1^ final concentration); Sigma-Aldrich].

### GC–MS Analysis

Gas chromatography–mass spectrometry (GC–MS) was done by a TRACE GC Ultra Gas Chromatograph coupled to an ITQ 900 GC-Ion Trap MS (both Thermo Scientific^TM^). The instrument was equipped with a Rtx^®^ -5MS column (30 m, iD 0.25, df 0.25 μm; Restek). Helium was used as carrier gas with a constant flow of 1 ml/min. 1 μl sample was injected (split less) for GC–MS analysis. The injector temperature was set to 250°C using a Trance & Focus GC-Liner. Before and after each injection the syringe was washed 10 times with 10 μl chloroform and hexane each. The oven program was: 3 min 80°C, ramp with 5°C per min up to 325°C, 2 min 325°C. The transfer line temperature was set at 250°C and the ion source at 220°C. Mass spectra were recorded at two scans per s from m/z 50 to 750. Electron energy was set to 70 eV. Raw data were converted to cdf-files by Xcalibur 2.0.7 software (Thermo) and uploaded to MeltDB (Neuweger et al., 2008). Ribitol was found to be absent in the samples and it was therefore possible to add it as an internal standard.

### Metabolite Normalization and Quantification of Selected Compounds

Samples were measured at least in biological triplicates due to the limited amount of embryonic tissue. Sample dry weight of about 5 mg was chosen in order to keep the detector response for high abundant compounds below the saturation limit as described earlier (Gorzolka et al., 2012). Peak detection in chromatograms was done with a signal to noise ratio of five followed by a multiple profiling to identify common MS patterns and to define TAGs as unknown compounds (Kopka, 2006). Supplementary Table S1 lists unknown or putatively identified compounds according to Sumner et al. (2007). Metabolites were identified according to the retention index (Kind et al., 2009) and mass spectra (MS) fitting to separately measured reference substances covering amino acids, mono- and di-saccharides, carbonic acids, and selected secondary metabolites. Normalization was performed on peak areas of characteristic compound masses (extracted ion chromatograms), normalized to ribitol (*m/z* 217) and material dry weight. Mass spectra of unknown metabolites were searched against the Golm-Metabolite Database (GMD; 2008) and NIST (2009) databases.

For absolute quantification, D-glucose (>99.5%, Carl Roth), D-sucrose (min. 99%, AppliChem), D-fructose (Sigma), γ-aminobutyric acid (GABA; SigmaUltra, min. 99%), L-proline (SigmaGrade, hydroxy-L-proline-free, Sigma) were measured in five dilution steps covering the observed concentration range.

### Statistical Analysis

For statistical comparison of the mean metabolite contents of the seed tissues and somatic embryos, the Tukey test was applied after log-transformation of the data, at *p* < 0.05, using the software R 3.1.0 (http://www.r-project.org).

## Results

### In the First Metabolite Profiles in *Cyclamen persicum* more than 200 Compounds were Detected and 52 were Identified

With this study, first insights into the primary metabolites in *C. persicum* seeds and somatic embryos were gained: the chromatograms of the four different tissues (Supplementary Figure S1) were dominated by the sucrose peaks, but showed several differences between the tissues. Overall, more than 200 metabolites were detected by GC–MS, out of which 52 were identified (**Table 1**). They were grouped into amino acids (20), sugars (10), sugar alcohols (4), organic acids (5), and others (13; **Table 1**). The time point 11 WAP was chosen for this comparative study, because at this time point, the embryo is fully developed and desiccation of the seed starts only afterwards (Schmidt et al., 2006). Moreover, this time point had also been selected for previous proteomic studies of embryos (Rode et al., 2011) and endosperm (Mwangi et al., 2013).

**Table 1:** Metabolites identified by gas chromatography-mass spectrometry in hydrophilic extracts of seed tissues and somatic embryos of *Cyclamen persicum*. *m/z* values in parentheses indicate the selective ions used for quantification.

Amino acids	Sugars	Sugar alcohols	Organic acids	Others
Alanine (116)	Arabinose (217, 307)	Glycerate (189, 192)	*Cis*-aconitate (229)	Adenine (264)
γ Aminobutyric acid (GABA) (174, 304)	Fructose (307)	Mannitol (217, 319)	Citric acid (257)	Adenosine (236)
Arginine (157, 256)	Fructose 6-P (315)	*Myo*-inositol (305)	Fumarate (245)	Catechin (368)
Asparagine (231)	Galactose (319)	*Myo*-inositol-P (318)	a-Hydroxyglutarate (203, 247)	Epicatechin (368)
Aspartate (100, 188, 232)	Glucose (319)		Malic acid (245, 307)	Ethanolamine (174)^∗^
Cysteine (220)	Glucose 6-P (387)			Gluconate-1,5-lactone (129, 220)^∗^
Glutamate (230, 246)	Raffinose (361)			Gluconate (333)
Glutamine (155)	Ribose (217)			Palmitic acid (117)^∗^
Glycine (174)	Sucrose (361)			Shikimate (204)
Homoserine (218)	Xylose (217, 307)			Stearic acid (185)^∗^
Isoleucine (158)				Thymine (255)^∗^
Leucine (158)				Uracil (255, 241)
Lysine (156)				Urea (189)
Methionine (176)				
Proline (142)				
Serine (204)				
Threonine (101)				
Tryptophan (202)				
Tyrosine (218)				
Valine (144)				

^*^only one data set available.

*Significant differences between somatic and zygotic embryos in both data sets (2012 and 2014) are indicated by the following color code:*

*Light yellow: >1.5 times in zygotic embryos.*

*Dark yellow: >5.0 times in zygotic embryos.*

*Light blue: >1.5 times in somatic embryos.*

*Dark blue: >5.0 times in somatic embryos.*

*Green frame: high content in testa.*

### Metabolite Profiles allow a Clear Separation of the Seed Tissues and Somatic Embryos

The 52 identified metabolites were relatively quantified for both sets of data (analyses run in 2012 and 2014) and were found to be consistent regarding the relation of the abundance in the different tissues in most cases (Supplementary Table S2, Supplementary Figure S2).

**FIGURE 2:**
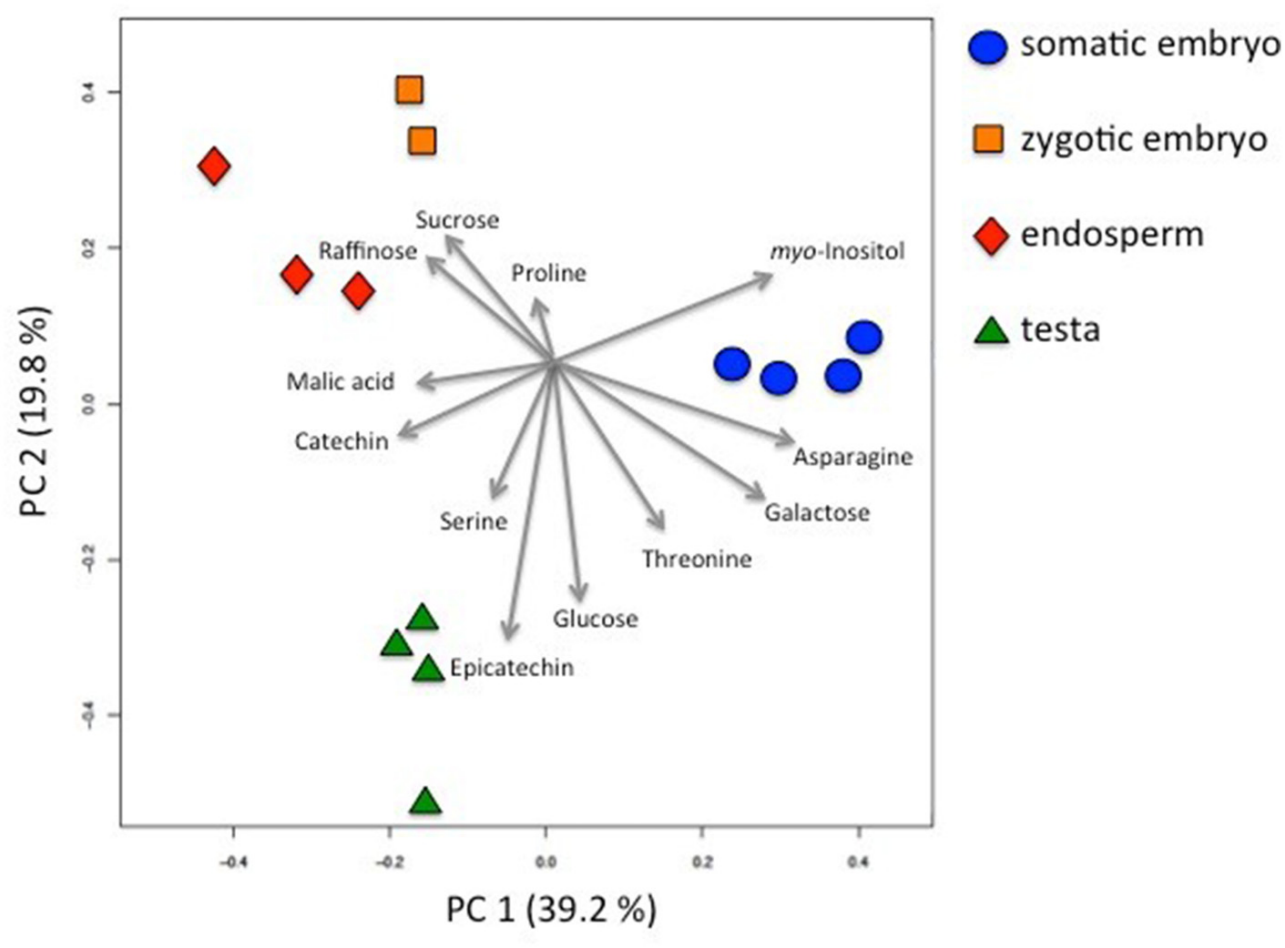
**Principal component analysis (PCA) of somatic embryos and different seed tissues of *C. persicum* on metabolite profile data gained from samples of 2014.** Gray arrows indicate the loading plots showing the contribution of the selected compounds to the principal compound 1 or 2. The metabolome of zygotic embryos and endosperm tissues showed the highest similarity, although also these samples are well-separated. Somatic embryos and the other samples are separated by the first principal compound, while the other samples are separated by the second principal compound.

A principal component analysis (PCA) calculated for the data obtained in 2014 revealed a clear separation of the different tissues (**Figure 2**). Similar results were obtained for the data set from 2012 (Supplementary Figure S1). For the data of 2014, the first axis explaining 39.2% of the variance mainly disjoined somatic embryos from seed tissues with *myo*-inositol, asparagine, galactose, catechin, malic acid, and threonine providing the strongest contribution to this first component. Endosperm and zygotic embryos were found to be most similar, while the second axis leads to a clear separation of testa metabolite profiles, driven mainly by epicatechin, serine, threonine, and glucose. Proline and sucrose were major compounds contributing to the separation of the zygotic embryos from the other tissues (**Figure 2**). When comparing the PCAs of both data sets, the principal separation and relations were very similar, and also the major metabolites contributing to the separation were found back in both years. Exceptions were galactose, malic acid, and raffinose, which only showed up in the PCA of 2014, but not in 2012.

### Metabolites of High Abundance in the Testa and the Endosperm

**FIGURE 3:**
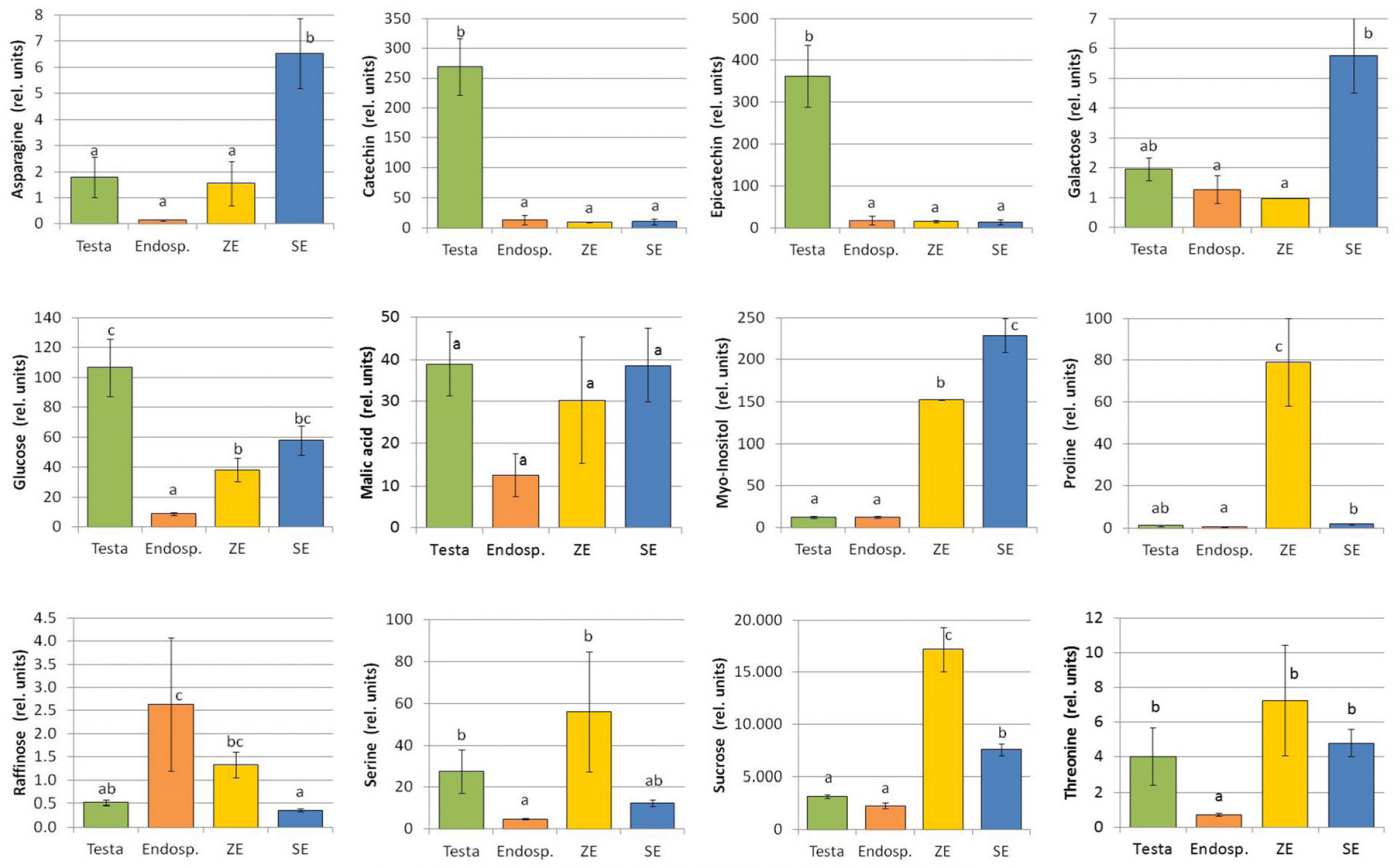
**Metabolite content of different seed tissues and somatic embryos of *C. persicum*.** Values are means and standard errors of the mean [*n* = 4; 3; 2; 4 for testa, endosperm, zygotic embryos (ZEs) and somatic embryos (SEs), respectively] of data gained from samples of 2014. Statistical significance tests (*p* < 0.05) were performed using the Tukey test. The selection of metabolites shown here is based on the PCA (**Figure 2**).

The testa stood out due to very high contents of catechin and epicatechin (**Figure 3**) that accumulated in the seed coat, especially in the samples analyzed in 2014 (Supplementary Table S2). In addition, fructose, glucose, and xylose concentrations were elevated in the testa compared to endosperm tissue (**Figure 3**, Supplementary Table S2). In contrast, the endosperm was found to be the tissue with the lowest contents of nearly all identified metabolites, except for raffinose.

### Different Metabolite Abundances in Somatic versus Zygotic Embryos

Since one of the major aims of this study was to identify differences in metabolite profiles between somatic and zygotic embryos in order to better understand possible causes of the described problems, these contrasts are of special importance and highlighted in **Table 1**. The most striking difference observed was the 40 fold higher concentration of proline determined in zygotic embryos when compared to their somatic counterparts (**Figure 3**). The quantification revealed 41 μg g^-1^ dry mass (DM) proline in zygotic embryos, but only about 1 μg g^-1^ DM in somatic embryos (**Table 2**). The second amino acid of significantly higher abundance in zygotic embryos was alanine. Smaller though significant differences with higher metabolite concentrations in zygotic embryos were observed for γ-aminobutyric acid (GABA) and glutamate. Sucrose concentrations were significantly higher in zygotic embryos (62566 μg g^-1^ DM (=6.3%) compared to 27660 μg g^-1^ DM (=2.8%) in somatic embryos, **Table 2**) despite the fact, that somatic embryos were cultivated on sucrose (30 g l^-1^) containing media. Also for raffinose, higher concentrations were observed in zygotic embryos although the differences were only significant for the data of 2014 (**Figure 3**).

**Table 2:** Concentrations of selected metabolites in different seed tissues and somatic embryos of *C. persicum* [μg g^-1^ DM].

	Testa	Endosperm	Zygotic embryo	Somatic embryo
Metabolite	*n* = 4	*n* = 3	*n* = 2	*n* = 4
Proline	0.63 + 0.12 ab	0.37 + 0.05 a	40.97 + 10.85 c	0.99 + 0.15 b
GABA	1.55 + 0.75 a	1.78 + 0.79 a	5.25 + 0.21 b	1.54 + 0.10 a
Fructose	1,750 + 345 b	120 + 24.2 a	974 + 658.2 b	1,213 + 251 b
Glucose	613 + 112 c	50.2 + 5.83 a	220 + 46 b	332 + 56 bc
Sucrose	11,439 + 673 a	8,212 + 965 a	62,566 + 6,251 c	27,660 + 1,808 b

*Pairwise comparison of means by the Tukey test at p ≤ 0.05 within rows: means indicated by the same letter did not differ significantly. Given are mean + standard error of the mean of n replicates.*

Metabolites of particularly higher concentrations in somatic embryos were tryptophan, galactose, adenosine, gluconate-1.5-lactone, shikimate, and especially ethanolamine (**Table 1**; **Figure 3**). Moreover, they contained higher contents of arabinose, fructose (in 2012, Supplementary Table S2), citric acid, glucose (highly significant in 2012), and *myo*-inositol (**Figure 3**).

In the chromatograms of zygotic embryos a hitherto unidentified peak at 50.04 min was present, that was much less abundant in endosperm and only very low in somatic embryos and testa (Supplementary Figure S1). The data base suggested this metabolite to be most likely a trisaccharide. However, due to different retention times we can exclude trehalose, cellobiose, turanose, verbascose, and stachyose as candidates for this chromatographic feature.

## Discussion

### Metabolite Composition of *C. persicum* Seed Tissues

Metabolite profiling of *C. persicum* seed tissues resulted in the identification of 52 identified compounds and more than 200 mass-time TAGs. Since no metabolite profiles were available for *C. persicum*, comparisons to literature are only possible for single compounds. Regarding seed development, strong differences within capsules can be observed due to subsequent development of the ∼150–200 ovules which are present in one flower (Reinhardt, 2006). Additional variation in development of seeds can be observed depending on the status of the mother plants. This most likely explains a major part of the variability in the relative quantification data (Supplementary Table S2). Moreover, variation between 2012 and 2014 may have derived from differences in culture conditions, for example due to seasonal variation in irradiation for the greenhouse-cultivated plants. Metabolite profiles reflect the physiological situation for a given time point, but are highly dynamic. Thus, in the following we will only focus on those metabolites that show similar relations of abundance for the investigated tissues in both years, 2012 and 2014. Only if differences were detected in both of these completely independent materials, they were regarded as relevant.

#### Testa/Seed Coat

After fertilization, seed coat formation derived from the integuments starts and nowadays the view on this organ is changing, because it is obviously more than just a protective layer (reviewed by Radchuk and Borisjuk, 2014). In the innermost layer of the testa, the endothelium synthesis of proanthocyanidins takes place, resulting after condensation to tannins and oxidation in the brown color of many seeds (Lepiniec et al., 2006). Consistent with this, catechin and epicatechin found in high concentrations in *C. persicum* testa (**Figure 3**) belong to the group of proanthocyanins which are the last intermediates before condensation into tannins takes place (Lepiniec et al., 2006). The testa of cyclamen seeds is white (**Figure 1B**), but turns yellow to brownish during ripening and dark brown when exposed to desiccation under air (Grey-Wilson, 2002). It has to be proven in future analyses how catechin and epicatechin in the *C. persicum* seed coat are biochemically modified during seed development and maturation. The functions of flavonoids in interaction of plants with their environment, in the defense response to biotic and abiotic stresses and in regulation of development have been described (see review of Lepiniec et al., 2006).

Cyclamen seeds are dispersed in nature by ants which are attracted by a sweet and sticky mucilage (Grey-Wilson, 2002). Thus, the high concentrations of glucose and fructose detected in the testa (**Table 2**, Supplementary Table S2) may reflect the attraction of the seed dispersers. Moreover, the seed coat has an important function in delivering nutrients from the mother plant to the developing seed across the apoplast (Radchuk and Borisjuk, 2014). This might explain the higher concentrations of all metabolites, except for arginine, palmitic acid, raffinose, and stearic acid in testa compared to endosperm tissue (Supplementary Table S2), although seed development would cease shortly after the sampling time 11WAP and desiccation would start. Future analyses of earlier stages could help to better understand metabolite transport in the developing *C. persicum* seed.

#### Endosperm

*Cyclamen persicum* seeds are dominated by a large endosperm (**Figure 1B**). Storage compounds described in cyclamen endosperm are at first xyloglucans, quite unusual storage carbohydrates detected in the cell walls of cyclamen endosperm (Braccini et al., 1995). In addition to the provision of energy, breakdown products of xyloglucans, oligosaccharides may exhibit growth stimulating effects during germination (Braccini et al., 1995). Storage proteins accumulate in *C. persicum* endosperm in protein bodies (Reinhardt, 2006). Proteins with data base matches to globulins were identified in endosperm and zygotic embryos (Winkelmann et al., 2006) and truncated enolases (Rode et al., 2011) were proposed to be novel forms of storage proteins for this species. The metabolite profiles of the present investigation revealed arginine, aspartate, alanine, glutamate, and serine to be the most abundant amino acids in *C. persicum* endosperm (Supplementary Table S2). Arginine and glutamine are major amino acids in storage proteins providing nitrogen and energy to the seedling during germination. The role of amino acids as an energy source in seeds was pointed out recently in a review of Galili et al. (2014): inner seed tissues exhibit hypoxic conditions in many species and also in *C. persicum* seeds (unpublished data). While in green seeds photosynthesis contributes to energy production in legume seeds for instance (Rolletschek et al., 2003), for non-green seeds, like *C. persicum*, amino acid metabolism is very important, and alanine seems to be especially important in this context (Rolletschek et al., 2011).

By Sudan black staining in sections of *C. persicum* seeds, lipids as third storage reserve were documented (Reinhardt, 2006). However, no information on the fatty acid composition is available, and proteomic studies of endosperm revealed only one enzyme, wax synthase catalyzing the transfer of an acyl chain from fatty acyl-coenzyme A (CoA) to a fatty alcohol, the final step in the synthesis of linear esters (Mwangi et al., 2013). In the present study palmitic and stearic acid were identified in *C. persicum* seeds, supporting the microscopic observations of lipid accumulation. Further analyses would be necessary to identify the fatty acid composition in this species.

The outstanding metabolite in all tissues under investigation was sucrose, which was detected in zygotic embryos in extremely high concentrations which were eight times higher than those found in the surrounding endosperm (**Table 2**). Reinhardt (2006) quantified sucrose, glucose, and fructose in ripe seeds of cyclamen by a photometric enzymatic assay and reported contents of about 15,000–28,000 μg g^-1^ fresh mass (FM) of sucrose, 5,500–10,800 μg g^-1^ FM fructose and 3,600–5,000 μg g^-1^ FM glucose. Assuming a low water content in ripe seeds and a proportion of 90–95% of the seed filled with endosperm, the figures for sucrose compare well to the values obtained in the present study, whereas fructose and glucose concentrations were lower in our analyses. Metabolite analyses in black soybean seeds of different maturation stages revealed an increase in sucrose concentration followed by a decrease late at seed maturity, while the monosaccharides glucose and fructose gradually decreased (Lee et al., 2013). The oligosaccharides raffinose and verbascose increased with seed maturation (Lee et al., 2013). Trisaccharides of the raffinose family, but also sucrose and *myo*-inositol, contribute to desiccation tolerance which is important for the last phases of seed maturation (Obendorf, 1997). Also, in the present study raffinose was detected in higher concentrations in the endosperm and in zygotic embryos compared to testa and somatic embryos (**Figure 3**). Besides their role in synthesis of storage compounds, sugars have important functions in regulating cell division, differentiation, and seed development (Weber et al., 1998), so temporal and spatial analyses would be essential to understand their way of action and to mimic their function when designing an artificial endosperm. One first approach can be the incorporation of raffinose in a maturation medium for somatic embryos.

### Pronounced Differences in Metabolite Profiles between Somatic and Zygotic Embryos

The comparison of the metabolite profiles of somatic and zygotic embryos will help us to understand the differences which should be linked to some of the problems observed for somatic embryos. This should enable the development of culture conditions and media compositions that result in somatic embryos of high quality resembling their zygotic counterparts. Comparisons of metabolite compositions of both embryo types are scarce. For cocoa, mature zygotic embryos contained more raffinose and stachyose but less sucrose, xylose and rhamnose than somatic embryos (Alemanno et al., 1997). Norway spruce mature zygotic embryos accumulated raffinose and stachyose, while somatic embryos did not (Gösslova et al., 2001). For the Brazilian fruit tree *Acca sellowiana* higher contents of sucrose, fructose, *myo*-inositol and raffinose were detected in zygotic embryos, but higher starch contents in somatic embryos (Pescador et al., 2008). Also in pea, soluble sugar composition differed between zygotic and somatic embryos with sucrose, galactinol, raffinose, verbascose, and stachyose being the prominent sugars in mature zygotic embryos. In contrast, among the soluble sugars in pea somatic embryos which contained much lower total amounts, fructose, glucose, *myo*-inositol, sucrose, raffinose, and galactinol were identified, but stachyose and verbascose were absent (Gorska-Koplinska et al., 2010). Interestingly, the carbohydrate profiles of misshaped somatic embryos differed from those of normal embryos (Gorska-Koplinska et al., 2010).

Defining a comparable stage for somatic and zygotic embryos is very difficult. In this study, the morphological stage was chosen as criterium, and both embryo types were in the torpedo stage at a time point before desiccation would start for zygotic embryos. For somatic embryos which do not undergo a growth arrest in our system (Schmidt et al., 2006) this stage was the last one before the physiological and morphological transition by the starting germination process would occur. Thus, although both were in a comparable morphological stage, their physiological status might have been different. Accordingly, part of the differences found in the metabolite profiles discussed below might have been caused by the different physiological states. In addition, variation in the micro-environment of different Petri dishes and also between different cell lines of the same genotype is also typically observed in tissue culture and might have contributed to deviations between the two years and different measurements within one year.

#### Metabolites of Significantly Higher Abundance in Zygotic Embryos

The most striking difference between both types of embryos was the 40-fold higher content of proline in zygotic embryos (**Figure 3**; **Table 2**). The role of proline in stress defense is well-known where it is functioning as molecular chaperone, as reactive oxygen species scavenger, as compatible solute, as well as in balancing pH and redox state (Verbruggen and Hermans, 2008). It might also serve as a nitrogen source in seeds. In plants, proline is synthesized mainly if not exclusively from glutamate (Mattioli et al., 2009a). Interestingly, evidence of a further function of proline in regulation of development comes from studies of *Arabidopsis* mutants in the proline biosynthesis genes *pyrroline-5-carboxylate synthetase* (*P5CS1* and *P5CS2*): high expression levels in the developing embryos suggest an important role of proline in early embryogenesis, and here mainly in cell division and meristem formation (Mattioli et al., 2009b). Thus, the observed high proline concentrations in *C. persicum* zygotic embryos could point to a high level of stress protection or also to an additional support of embryo development by proline in this late phase of embryogenesis.

GABA (γ-aminobutyric acid) has received attention in animals due to its involvement in neurotransmission, whereas in plants, for a long time, it has been regarded as metabolite without further roles (Bouché and Fromm, 2004). Bouché and Fromm (2004) list the possible roles of GABA and the GABA shunt that show similarities to proline functions: contribution to C:N balance, regulation of pH, protection against oxidative stress, osmoregulation, signaling molecule. In a methodological paper dealing with the detection of temporal and spatial GABA concentration within plant tissues, a high concentration of GABA was detected in seeds of *Solanum melanogaster* (Goto-Inoue et al., 2010). Possibly the higher GABA concentrations in zygotic embryos of *C. persicum* compared to somatic embryos (**Table 2**) present an aspect acting in the same direction as proline.

Zygotic embryos contained significantly higher amounts of sucrose and raffinose than their somatic counterparts. Sucrose was shown to be an important seed metabolite not only for providing energy, but also as signal that regulates embryo development (Weber et al., 1998). Moreover, like raffinose sucrose is contributing to desiccation tolerance of the embryo in later phases of seed development (Obendorf, 1997).

Unfortunately, the putative trisaccharide present in zygotic embryo histograms of *C. persicum* could not be identified until now. Trehalose, cellobiose, turanose, verbascose, and stachyose could be excluded by separate GC–MS analyses. Rothe et al. (1999) identified planteose, an unusual trisaccharide composed of glucose, fructose, and galactose in *C. persicum* phloem sap. Besides sucrose it was one of the main carbohydrates in the sap. Rothe et al. (1999) also quantified sucrose, fructose, and glucose in different *C. persicum* organs and always found that the concentrations of fructose were higher than those of glucose as was also observed in the seed tissues analyzed here (**Table 2**).

*Myo*-Inositol-P was detected in significantly higher concent rations in zygotic embryos, whereas somatic embryos contained higher *myo*-inositol concentrations (significant only in data gained in 2014; **Figure 3**, Supplementary Table S2). Since *myo*-inositol was offered to the culture medium (100 mg l^-1^) the higher contents of somatic embryos might reflect this fact. Inositol phosphates are cellular signals mobilizing calcium and are involved in phosphorus storage of seeds as phytic acid as well as membrane lipid metabolism (Shears, 1998). In maize, phytic acid has been shown to confer tolerance to oxidative stress with high concentrations found in the zygotic embryo (Doria et al., 2009).

#### Metabolites of Significant Higher Abundance in Somatic Embryos

Among the metabolites of higher abundance in somatic embryos, i.e., tryptophan, galactose, adenosine, gluconate-1.5-lactone, shikimate, arabinose, fructose, citric acid, glucose, *myo*-inositol, and ethanolamine, the latter showed the most drastic difference. Unfortunately, it was not possible to quantify ethanolamine in the samples taken in 2012 due to traces also detected in the blanks. Ethanolamine in plants is synthesized from serine (Rontein et al., 2001) and plays a role in the synthesis of choline, phosphatidylethanolamine, and phosphatidylcholine that are important for cell membranes (Kwon et al., 2012). Mutants in serine decarboxylase showed developmental defects, such as necroses on leaves, malformed inflorescences, earlier flowering, and sterility, that could be restored by supplementation with ethanolamine indicating a role in plant development as well (Kwon et al., 2012). Ethanolamine has also been detected as significant metabolite in late phases of somatic embryogenesis in *Picea abies* (Businge et al., 2012).

Somatic embryos apparently had a higher metabolic activity as displayed by adenine, glucose, fructose, citric acid, and shikimate that were found to be more abundant than in zygotic embryos (**Table 2**, Supplementary Table S2). These observations can be correlated to the results of proteomic comparisons of both types of *C. persicum* embryos, in which also a higher metabolic activity of somatic embryos was observed indicating the lack of growth arrest at the end of embryo development. By higher sucrose concentrations and a treatment with abscisic acid, the metabolic activity of somatic embryos was reduced resulting in a protein pattern which resembled more that of zygotic embryos (Rode et al., 2012).

The increased contents of galactose and arabinose in somatic embryos could suggest a connection to arabinogalactan proteins (AGPs). AGPs are important molecules in cell-cell-communication and were shown to be involved in the induction and development of somatic embryos (van Hengel et al., 2001). Also in *C. persicum*, AGPs from carrot cultures stimulated somatic embryogenesis (Kreuger et al., 1995). In many systems, somatic embryos are surrounded by a so-called extracellular surface matrix network (ESMN), in which AGPs have been detected (Steinmacher et al., 2012). Also in *C. persicum* we have microscopic observations (unpublished data) suggesting the presence of an ESMN, which forms an envelope around the somatic embryos and most likely was harvested with the samples for the metabolite analyses in the present study. The direct detection of AGPs by specific antibodies should follow in future studies.

## Conclusion

First insights into metabolites present in different compartments of *C. persicum* seeds have been gained within this study. In future, observations during seed development are needed in order to better understand the time course and dynamics of accumulation of different metabolites. The identification of the yet unknown oligosaccharide found in the zygotic embryos could shed light into the unusual carbohydrate metabolism of *C. persicum* seeds. From the comparison of zygotic and somatic embryos we can conclude that zygotic embryos accumulate an arsenal of stress defense metabolites, such as proline, GABA and *myo*-inositol phosphate, that are found in much lower concentrations in somatic embryos. In contrast, somatic embryos are characterized by greater metabolic activity. An approach for the optimization of somatic embryogenesis could be the design of an appropriate maturation medium, which should lead to a clear separation of the differentiation phase and the later germination phase, and could include the addition of sucrose, raffinose, proline, GABA, and glutamate.

## Conflict of Interest Statement

The authors declare that the research was conducted in the absence of any commercial or financial relationships that could be construed as a potential conflict of interest.
