# Supplementary material for: Metabolite profiling of somatic embryos of Cyclamen persicum in comparison to zygotic embryos, endosperm, and testa
Source: Front Plant Sci. 2015 Aug 4;6:597. doi: 10.3389/fpls.2015.00597 (PMC4523879; doi:10.3389/fpls.2015.00597)
Supplement: Supplementary file 1 [file Table_1.DOCX]

**Supplementary Table 1**: Metabolite concentrations (relative units) of unknown or putatively identified compounds in different seed tissues and somatic embryos of *Cyclamen persicum* in samples harvested in 2014. Analytes were classified according to Sumner et al. (2007). Compounds according to level 1 identification (Sumner et al. 2007) are listed in Supplementary Table 2. Given are means and standard errors of n replicates.

| Metabolite | Testa  (n = 4) | Endosperm  (n = 3) | Zygotic embryos (n = 2) | Somatic embryos (n = 4) |
| --- | --- | --- | --- | --- |
|  |  |  |  |  |
| Galactinol ^2^ | 9.21 ± 1.34 | 0.75 ± 0.18 | 15.55 ± 2.31 | 17.77 ± 4.74 |
| Galactinol-2 ^2^ | 4.67 ± 0.41 | 17.02 ± 7.07 | 34.23 ± 6.51 | 96.64 ± 13.83 |
| Gallic acid ^2^ | 143.17 ± 29.57 | 46.53 ± 41.77 | 34.76 ± 0.95 | 96.32 ± 16.22 |
| Glycerol ^2^ | 13.34 ± 0.89 | 12.55 ± 3.35 | 30.17 ± 6.30 | 11.25 ± 1.46 |
| Maltose/Isomaltose ^2^ | 0.32 ± 0.10 | 0.02 ± 0.02 | 0.38 ± 0.37 | 23.76 ± 8.34 |
| N-acetyl-L-glutamic acid ^2^ | n.d. | 0.92 ± 0.86 | 0.25 ± 0.01 | 0.15 ± 0.04 |
| Octadecanoid acid methyl ester ^2^ | 0.44 ± 0.08 | 1.64 ± 1.01 | 2.25 ± 1.59 | 0.31 ± 0.05 |
| Oxalic acid ^2^ | 0.70 ± 0.11 | 0.63 ± 0.31 | 1.54 ± 0.11 | 0.69 ± 0.12 |
| Phosphoric acid ^2^ | 71.07 ± 7.04 | 46.61 ± 17.36 | 228.64 ± 23.51 | 116.35 ± 5.87 |
| Threonic acid ^2^ | 2.82 ± 0.57 | 0.19 ± 0.05 | 3.91 ± 2.52 | 2.50 ± 0.33 |
| Uric acid ^2^ | 288.34 ± 35.63 | 6.19 ± 6.08 | 2.12 ± 0.62 | 9.38 ± 3.82 |
|  |  |  |  |  |
| Unknown (organic acid) ^3^ | 3.82 ± 0.84 | 2.50 ± 0.24 | 10.64 ± 2.22 | 2.84 ± 0.55 |
| Unknown (4-hydroxy benzoic acid-like) ^3^ | 1.31 ± 0.15 | 0.56 ± 0.51 | 0.36 ± 0.06 | 0.55 ± 0.24 |
| Unknown (4-hydroxy benzoic acid-like) ^3^ | 2.21 ± 0.46 | 0.86 ± 0.60 | 2.63 ± 0.46 | 13.69 ± 2.04 |
| Unknown (sugar) ^3^ | 9.78 ± 3.07 | 2.64 ± 1.38 | 26.94 ± 19.14 | 0.56 ± 0.26 |
| Unknown (sugar) ^3^ | 0.90 ± 0.18 | 0.15 ± 0.03 | 3.14 ± 0.35 | 1.02 ± 0.12 |
| Unknown (sugar) ^3^ | 21.12 ± 5.51 | 66.04 ± 16.75 | 1270.32 ± 545.57 | 30.59 ± 10.05 |
|  |  |  |  |  |
| Unknown-1 ^4^ | 0.85 ± 0.33 | 1.57 ± 0.77 | 6.32 ± 4.19 | 2.72 ± 0.95 |
| Unknown-2 ^4^ | 8.48 ± 0.85 | 15.65 ± 8.58 | 25.51 ± 6.90 | 13.56 ± 1.80 |
| Unknown-3 ^4^ | 1.47 ± 0.50 | 0.30 ± 0.09 | 1.37 ± 0.64 | 9.09 ± 2.14 |
| Unknown-4 ^4^ | 3.69 ± 0.39 | 1.18 ± 1.07 | 0.93 ± 0.06 | 1.49 ± 0.46 |
| Unknown-5 ^4^ | 0.15±0.01 | 0.13 ± 0.01 | 0.46 ± 0.16 | 0.21 ± 0.03 |
| Unknown-6 ^4^ | 51.08 ± 7.08 | 11.17 ± 4.76 | 60.34 ± 31.15 | 15.16 ± 3.69 |
| Unknown-7 ^4^ | 0.57 ± 0.34 | 0.20 ± 0.08 | 1.48 ± 0.02 | 85.14 ± 39.86 |
| Unknown-8 ^4^ | 1.47 ± 0.47 | 0.22 ± 0.05 | 7.45 ± 1.51 | 67.75 ± 5.67 |
| Unknown-9 ^4^ | 1.97 ± 0.60 | 2.10 ± 0.21 | 8.48 ± 0.66 | 3.61 ± 0.63 |
| Unknown-10 ^4^ | 1.90 ± 0.23 | 1.67 ± 0.47 | 5.95 ± 1.56 | 4.28 ± 0.69 |
| Unknown-11 ^4^ | 3.51 ± 0.36 | 5.07 ± 3.19 | 8.35 ± 2.51 | 0.99 ± 0.50 |

^2^ Putatively annotated compound, identified by comparison with spectral data libraries

^3^ Putatively characterized compound, with spectral similarities to certain chemical class

^4^ Unknown compound, unclassified metabolite
