# Supplementary material for: Metabolite profiling of somatic embryos of Cyclamen persicum in comparison to zygotic embryos, endosperm, and testa
Source: Front Plant Sci. 2015 Aug 4;6:597. doi: 10.3389/fpls.2015.00597 (PMC4523879; doi:10.3389/fpls.2015.00597)
Supplement: Supplementary file 2 [file Table_2.DOCX]

**Supplementary Table 2**: Metabolite concentrations (relative units) in different seed tissues and somatic embryos of *Cyclamen persicum* analysed in 2012 and 2014. *m/z* values in parentheses indicate the selective ions used for quantification. Given are means and standard errors of n replicates. All compounds were identified by measurements of the corresponding reference standards according to level 1 identification as published by Sumner et al. (2007).

|  | **Testa** | | **Endosperm** | | **Zygotic embryo** | | **Somatic embryo** | |
| --- | --- | --- | --- | --- | --- | --- | --- | --- |
| **Metabolite** | 2012 (n = 3) | 2014 (n = 4) | 2012 (n = 3) | 2014 (n = 3) | 2012 (n = 3) | 2014 (n = 2) | 2012 (n = 3) | 2014 (n = 4) |
| Adenine (264) | 0.25 + 0.02 | 1.57 + 0.59 | 0.05 + 0.01 | 2.92 + 1.68 | 0.21 + 0.06 | 5.15 + 0,88 | 0.65 + 0.09 | 2.00 + 0.30 |
| Adenosine (236) | 0.01 + 0.00 | 0.10 + 0.02 | 0.01 + 0.00 | 0.06 + 0.01 | 0.02 + 0.01 | 0.26 + 0.00 | 0.64 + 0.08 | 1.47 + 0.18 |
| a-Hydroxyglutarate (203.247) | 0.15 + 0.06 | 0.20 + 0.05 | 0.22 + 0.16 | 0.01 + 0.00 | 3.45 + 0.30 | 0.29 + 0.21 | 8.00 + 0.86 | 0.18 + 0.03 |
| Alanine (116) | 142.37 + 35.11 | 27.10 + 9.46 | 11.97 + 1.72 | 9.47 + 1.26 | 56.63 + 12.95 | 58.78 + 28.56 | 9.98 + 1.49 | 2.41 + 0.27 |
| Arabinose (217.307) | 0.78 + 0.16 | 0.56 + 0.06 | 0.25 + 0.04 | 0.29 + 0.03 | 0.46 + 0.03 | 1.16 + 0.11 | 1.22 + 0.25 | 5.70 + 1.34 |
| Arginine (157.256) | 10.30 + 1.44 | 6.81+ 1.79 | 20.71 + 3.74 | 15.01 + 3.77 | 37.99 + 1.09 | 75.53 + 12.78 | 49.89 + 15.16 | 44.33 + 5.27 |
| Asparagine (231) | 6.80 + 2.08 | 1.80 + 0.77 | 0.17 + 0.02 | 0.15 + 0.03 | 2.19 + 0.93 | 1.56 + 0.85 | 1.39 + 0.54 | 6.53 + 1.34 |
| Aspartate (100.188.232) | 40.45 + 3.95 | 16.31 + 8.40 | 22.40 + 5.52 | 3.31 + 0.22 | 51.18 + 7.27 | 35.95 + 6.26 | 49.17 + 4.10 | 53.51 + 3.47 |
| Catechin (368) | 21.50 + 1.95 | 269.02 + 47.54 | 12.04 + 0.68 | 13.78 + 8.04 | 4.28 + 2.07 | 9.84 + 0.93 | 0.20 + 0.10 | 10.37 + 4.86 |
| Cis-Aconitate (229) | 0.47+ 0.08 | 0.02 + 0.01 | 0.21 + 0.07 | 0.00 + 0.00 | 0.72 + 0.04 | 0.02 + 0.11 | 1.35 + 0.60 | 0.03 + 0.01 |
| Citric acid (257) | 9.05 + 2.11 | 21.26 + 3.35 | 6.04 + 0.79 | 3.76 + 0.86 | 29.20 + 0.67 | 100.97 + 9.07 | 42.12 + 17.54 | 285.93 + 41.15 |
| Cysteine (220) | 0.29 + 0.10 | 0.01 + 0.00 | 0.06 + 0.01 | 0.01 + 0.00 | 0.38 + 0.09 | 0.05 + 0.01 | 1.47 + 0.21 | 0.26 + 0.06 |
| Epicatechin (368) | 14.86 + 2.17 | 361.96 + 74.36 | 14.34 + 0.91 | 18.23 + 10.65 | 5.42 + 2.36 | 15.38 + 2.45 | 0.69 + 0.19 | 14.12 + 6.28 |
| Ethanolamine (174) | n.d. | 3.81 + 1.10 | n.d. | 4.28 + 1.13 | n.d. | 10.88 + 1.06 | n.d. | 395.45 + 125.73 |
| Fructose (307) | 100.50 + 25.09 | 65.38 + 11.11 | 9.60 + 1.61 | 4.90 + 1.16 | 17.71 + 6.38 | 35.32 + 23.83 | 110.90 + 19.02 | 42.06 + 9.35 |
| Fructose-6-P (315) | 1.15 + 0.18 | 0.95 + 0.18 | 1.52 + 0.37 | 0.35 + 0.07 | 2.49 + 0.55 | 1.34 + 0.52 | 2.66 + 1.08 | 0.78 + 0.07 |
| Fumarate (245) | 0.68 + 0.23 | 1.31 + 0.20 | 0.49 + 0.11 | 0.55 + 0.13 | 0.43 + 0.04 | 1.42 + 0.10 | 1.47 + 0.50 | 0.78 + 0.07 |
| γ-Amino butyric acid (GABA)(174.304) | 5.07 + 1.64 | 1.38 + 0.67 | 0.84 + 0.24 | 1.56 + 0.69 | 8.39 + 1.41 | 4.54 + 0.08 | 1.82 + 0.19 | 1.31 + 0.11 |
| Galactose (319) | 3.01 + 0.61 | 1.96 + 0.38 | 1.46 + 0.23 | 1.27 + 0.47 | 0.83 + 0.27 | 0.98 + 0.00 | 3.18 + 0.15 | 5.77 + 1.26 |
| Gluconat-1.5-lacton (129.220) | 2.64 + 1.32 | n.d. | 0.75 + 0.12 | n.d. | 2.62 + 0.44 | n.d. | 15.37 + 2.02 | n.d. |
| Gluconate (333) | 0.35 + 0.04 | 0.15 + 0.02 | 0.39 + 0.07 | 0.05+ 0.02 | 3.35 + 0.52 | 0.73 + 0.16 | 19.85 + 1.73 | 1.74 + 0.29 |
| Glucose (319) | 29.60 + 4.85 | 106.61 + 19.40 | 5.66 + 0.59 | 8.74 + 1.01 | 8.64 + 2.72 | 38.27 + 8.02 | 73.05 + 8.13 | 57.69 + 9.64 |
| Glucose-6-P (387) | 1.65 + 0.24 | 1.50 + 0.28 | 2.39 + 0.53 | 0.70+ 0.12 | 3.22 + 0.75 | 3.30 + 1.17 | 5.10 + 2.03 | 1.46 + 0.04 |
| Glutamate (230.246) | 21.49 + 5.59 | 43.22 + 6.05 | 11.37 + 1.72 | 10.62 + 0.89 | 67.83 + 4.07 | 107.34 + 0.49 | 44.61 + 6.36 | 58.56 + 5.09 |
|  | **Testa** | | **Endosperm** | | **Zygotic embryo** | | **Somatic embryo** | |
| **Metabolite** | 2012 (n = 3) | 2014 (n = 4) | 2012 (n = 3) | 2014 (n = 3) | 2012 (n = 3) | 2014 (n = 2) | 2012 (n = 3) | 2014 (n = 4) |
| Glutamine (155) | 0.76 + 0.25 | 0.76 + 0.17 | 0.07 + 0.01 | 0.11 + 0.09 | 0.38 + 0.19 | 0.39 + 0.22 | 0.65 + 0.29 | 2.21 + 0.52 |
| Glycerate (189.192) | 0.91 + 0.22 | 0.84 + 0.10 | 0.50 + 0.07 | 0.36 + 0.04 | 0.70 + 0.26 | 0.86 + 0.07 | 2.27 + 0.23 | 0.79 + 0.11 |
| Glycine (174) | 5.38 + 1.32 | 1.40 + 0.26 | 1.00 + 0.13 | 0.74 + 0.05 | 6.37 + 1.11 | 4.88 + 1.04 | 5.07 + 0.17 | 2.27 + 0.30 |
| Homoserine (218) | 3.17 + 0.21 | 0.16 + 0.04 | 2.20 + 0.21 | 0.05 + 0.02 | 3.15 + 0.10 | 0.33 + 0.20 | 9.62 + 3.13 | 0.11 + 0.02 |
| Isoleucine (158) | 5.46 + 2.11 | 1.30 + 0.38 | 0.52 + 0.11 | 0.40 + 0.04 | 1.91 + 0.58 | 2.77 + 0.85 | 6.65 + 2.85 | 3.63 + 0.44 |
| Leucine (158) | 2.59 + 0.39 | 1.62 + 0.32 | 0.73 + 0.30 | 1.07 + 0.12 | 2.41 + 0.74 | 4.09 + 0.39 | 5.28 + 1.30 | 3.35 + 0.35 |
| Lysine (156) | 3.37 + 0.69 | 1.30 + 0.28 | 4.99 + 1.13 | 1.27 + 0.35 | 26.33 + 1.58 | 17.79 + 0.46 | 37.72 + 11.72 | 13.68 + 3.05 |
| Malic acid (245.307) | 22.21 + 4.81 | 38.94 + 7.57 | 13.33 + 2.10 | 12.54 + 5.07 | 16.28 + 0.28 | 30.33 + 14.97 | 22.75 + 4.32 | 38.55 + 8.73 |
| Mannitol (217.319) | 2.81 + 0.07 | 2.35 + 0.43 | 1.98 + 0.16 | 1.02 + 0.19 | 4.04 + 0.28 | 3.39 + 0.31 | 6.97 + 1.79 | 3.46 + 0.45 |
| Methionine (176) | 0.09 + 0.07 | 0.22 + 0.08 | 0.03 + 0.01 | 0.11 + 0.01 | 0.42 + 0.25 | 1.32 + 0.36 | 7.27 + 3.50 | 2.61 + 0.34 |
| *Myo*-Inositol (305) | 7.75 + 0.55 | 12.21 + 1.07 | 12.30 + 0.74 | 12.39 + 1.30 | 42.93 + 2.32 | 152.76 + 0.44 | 53.21 + 5.78 | 228.80 + 20.28 |
| *Myo*-Inositol-P (318) | 1.34 + 0.26 | 3.08 + 0.68 | 1.22 + 0.14 | 1.37 + 0.24 | 9.41 + 0.23 | 30.77 + 6.95 | 6.31 + 1.47 | 8.99 + 1.14 |
| Palmitic acid (117) | n.d. | 103.94 + 17.56 | n.d. | 128.18 + 35.88 | n.d. | 115.95 + 26.91 | n.d. | 96.71 + 11.91 |
| Proline (142) | 1.54 + 0.22 | 1.21 + 0.23 | 0.68 + 0.26 | 0.72 + 0.09 | 99.57 + 24.43 | 79.15 + 20.97 | 2.69 + 1.02 | 1.92 + 0.29 |
| Raffinose (361) | 28.35 + 4.35 | 0.52 + 0.06 | 76.94 + 3.79 | 2.64 + 1.43 | 47.13 + 21.81 | 1.33 + 0.28 | 19.15 + 2.97 | 0.37 + 0.03 |
| Ribose (217) | 2.35 + 0.69 | 1.02+ 0.17 | 0.79 + 0.17 | 0.53 + 0.23 | 1.19 + 0.24 | 0.80 + 0.11 | 0.89 + 0.05 | 1.53 + 0.20 |
| Serine (204) | 101.53 + 11.72 | 27.48 + 10.28 | 11.07 + 1.62 | 4.64 + 0.36 | 62.76 + 20.05 | 56.09 + 28.62 | 23.29 + 4.13 | 12.26 + 1.56 |
| Shikimate (204) | 5.60+ 1.67 | 4.35 + 0.73 | 1.77 + 0.49 | 0.24 + 0.08 | 0.61 + 0.32 | 0.98 + 0.43 | 7.38 + 2.26 | 15.42 + 1.82 |
| Stearic acid (185) | n.d. | 14.95 + 2.51 | n.d. | 20.77 + 7.53 | n.d. | 15.27 + 2.31 | n.d. | 12.21 + 1.06 |
| Sucrose (361) | 464.9 + 50.66 | 3143.6 + 178.55 | 728.4 + 68.74 | 2271.0 + 262.70 | 3281.5 + 180.56 | 17190.6 + 2108.84 | 2184.1 + 152.74 | 7607.5 + 565.95 |
| Threonine (101) | 13.31 + 3.03 | 4.03 + 1.62 | 1.71 + 0.29 | 0.71 + 0.07 | 8.21 + 1.86 | 7.25 + 3.18 | 3.88 + 0.80 | 4.79 + 0.78 |
| Thymine (255) | n.d. | 0.39 + 0.09 | n.d. | 0.32 + 0.07 | n.d. | 0.98 + 0.11 | n.d. | 0.37 + 0.06 |
| Tryptophan (202) | 0.25 + 0.14 | 0.41 + 0.14 | 0.27 + 0.02 | 0.45 + 0.16 | 0.85 + 0.38 | 1.91 + 1.04 | 21.48 + 4.10 | 11.65 + 1.58 |
| Tyrosine (218) | 4.22 + 1.33 | 1.24 + 0.29 | 0.52 + 0.11 | 0.36 + 0.13 | 1.69 + 0.47 | 2.71 + 0.79 | 4.25 + 0.34 | 9.52 + 2.60 |
| Uracil (255.241) | 2.17+ 1.07 | 6.23 + 0.71 | 0.45 + 0.11 | 3.18 + 0.59 | 0.37 + 0.14 | 8.01 + 1.49 | 0.28 + 0.14 | 2.68 + 0.22 |
| Urea (189) | 0.68 + 0.11 | 0.61 + 0.07 | 0.76 + 0.15 | 0.56 + 0.23 | 1.07 + 0.05 | 2.40 + 0.99 | 3.24 + 1.14 | 1.94 + 0.31 |
| Valine (144) | 16.74 + 4.93 | 3.12 + 1.04 | 1.75 + 0.24 | 0.96 + 0.09 | 7.41 + 1.20 | 6.80 + 2.25 | 8.43 + 2.48 | 6.88 + 0.95 |
| Xylose (217. 307) | 9.44 + 4.06 | 43.02 + 9.90 | 2.57 + 0.80 | 2.60 + 0.67 | 1.34 + 1.11 | 4.70 + 1.64 | 1.21 + 0.37 | 5.71 + 1.41 |

n.d. = not detected
