# Supplementary material for: Metabolite profiling of somatic embryos of Cyclamen persicum in comparison to zygotic embryos, endosperm, and testa
Source: Front Plant Sci. 2015 Aug 4;6:597. doi: 10.3389/fpls.2015.00597 (PMC4523879; doi:10.3389/fpls.2015.00597)
Supplement: Supplementary file 3 [file Image_1.PDF]

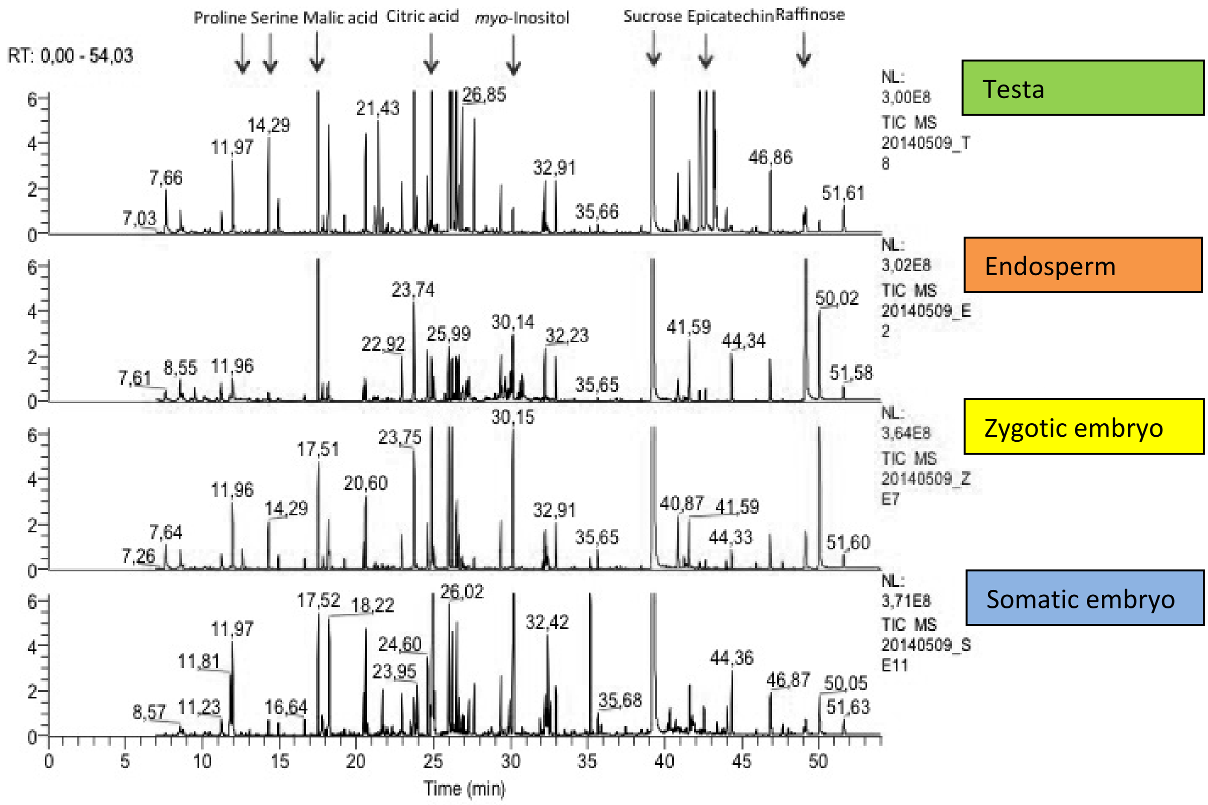

Supplementary Figure 1: Gas chromatography-mass spectrometry (GC-MS) based metabolite fingerprints of different seed tissues and somatic embryos of *Cyclamen persicum*. The total ion chromatogram (TIC) of methanolic extracts of testa, endosperm, zygotic embryos and somatic embryos resulted in the detection of up to 300 chromatographic signals. All samples showed characteristic differences in individual analytes.
