# Supplementary material for: Metabolite profiling of somatic embryos of Cyclamen persicum in comparison to zygotic embryos, endosperm, and testa
Source: Front Plant Sci. 2015 Aug 4;6:597. doi: 10.3389/fpls.2015.00597 (PMC4523879; doi:10.3389/fpls.2015.00597)
Supplement: Supplementary file 4 [file Image_2.PDF]

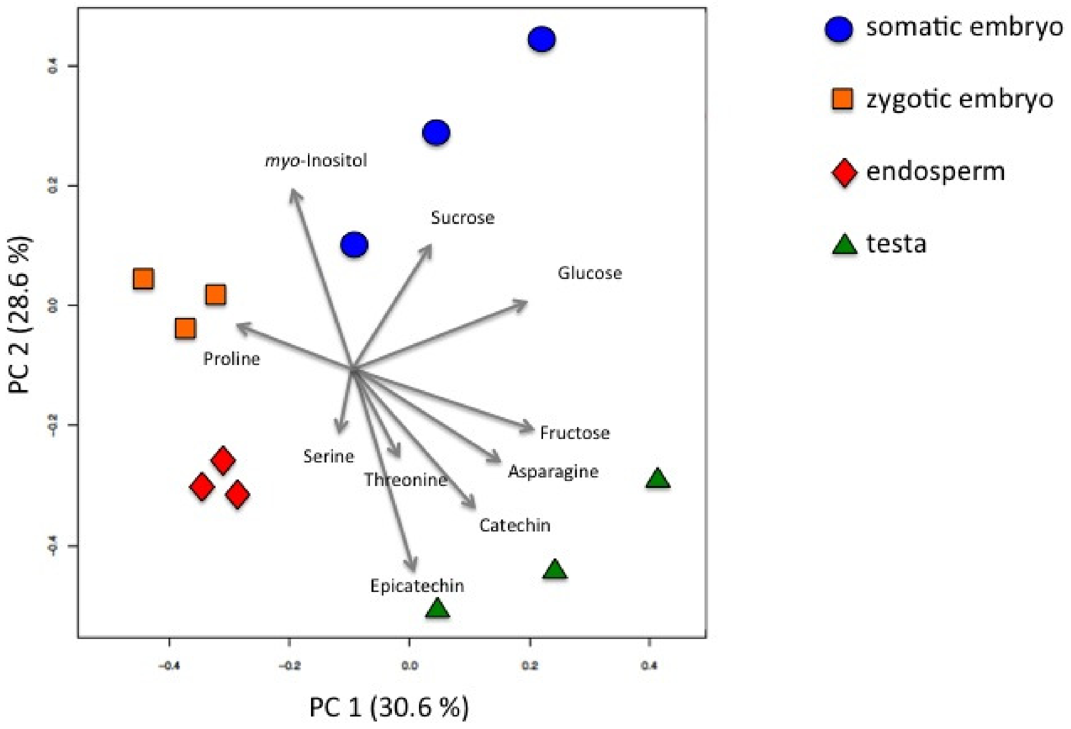

Supplementary Figure 2: Principal component analysis (PCA) of somatic embryos and different seed tissues of *Cyclamen persicum* on metabolite profile data gained from samples of 2012. Gray arrows indicate the loading plots showing the contribution of the selected compounds to the principal compound 1 or 2. The metabolome of zygotic embryos and endosperm tissues showed the highest similarity. Somatic embryos and the testa samples are separated by the first principal compound, while both types of embryos are separated from testa and endosperm samples by the second principal compound.
